# The effect of frailty should be considered in the management plan of older people with Type 2 diabetes

**DOI:** 10.4155/fsoa-2015-0016

**Published:** 2016-02-12

**Authors:** Ahmed H Abdelhafiz, Luan Koay, Alan J Sinclair

**Affiliations:** 1 Department of Geriatric Medicine, Rotherham General Hospital, Moorgate Road, Rotherham, S60 2UD, UK; 2 Foundation for Diabetes Research in Older People, Diabetes Frail Ltd, Droitwich Spa, WR9 0QH, UK

**Keywords:** frailty, hypoglycemia, older people, Type 2 diabetes

## Abstract

The prevalence of diabetes is increasing especially in older age due to increased life expectancy. In old age, diabetes is associated with high comorbidity burden and increased prevalence of geriatric syndromes including frailty in addition to micro- and macro-vascular complications. The emergence of frailty may change the natural history of Type 2 diabetes from a progressive to a regressive course with increased risk of hypoglycemia. This may result in normalization of blood glucose levels and lead to a state of burnt-out diabetes in frail older people with significant weight loss. Although guidelines suggest relaxed glycemic control in frail elderly with diabetes, complete withdrawal of hypoglycemic medications may be necessary in these frail populations to reduce the risk of hypoglycemia.

## Abstract (lay abstract)

**Lay abstract:** Diabetes is a common disease especially in older people. Aging is associated with reduced appetite and reduced food intake due to lack of exercise and diminished energy consumption. In certain individuals this may be significant leading to weight loss, malnutrition and the development of frailty. As a result the doses of diabetes medications may be too strong for this frail population especially if they have organ dysfunction reducing medication clearance and increasing the risk of low blood glucose level events. Therefore, reduction or complete withdrawal of diabetes medications may be considered in this frail group.

**Figure 1.**
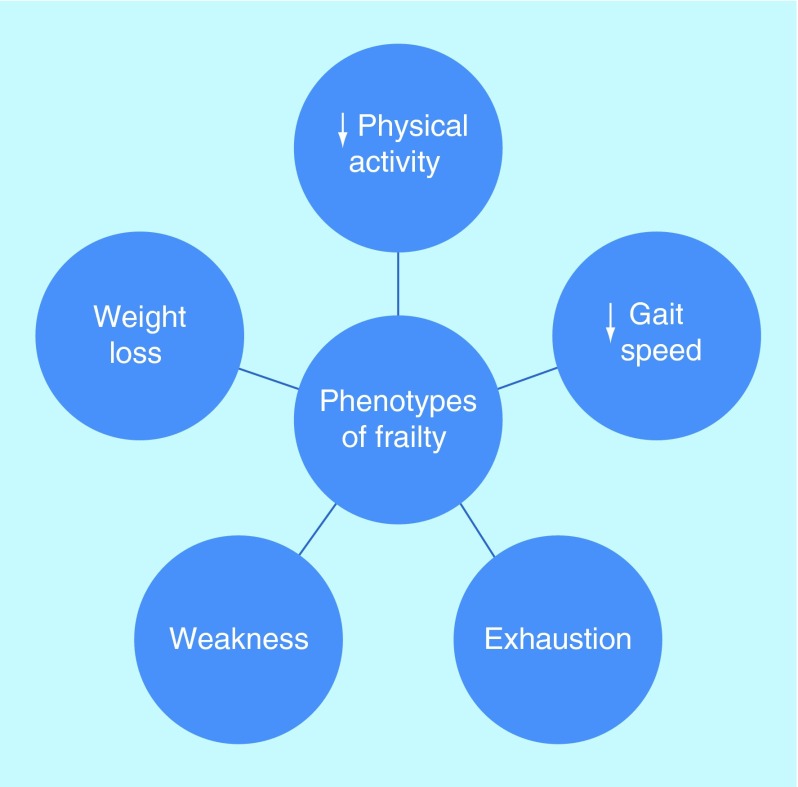
**Phenotypes of frailty.** Presence of 0–1 phenotype = not frail, two phenotypes = pre-frail, ≥three phenotypes = frail [5].

**Figure 2.**
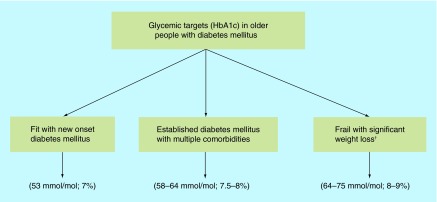
**Glycemic targets in older people with diabetes mellitus.** ^†^Short-term targets of daily blood glucose monitoring between >4 but <15 mmol/l are more relevant than the long-term HbA1c due to the limited life expectancy in this population group.

**Figure 3.**
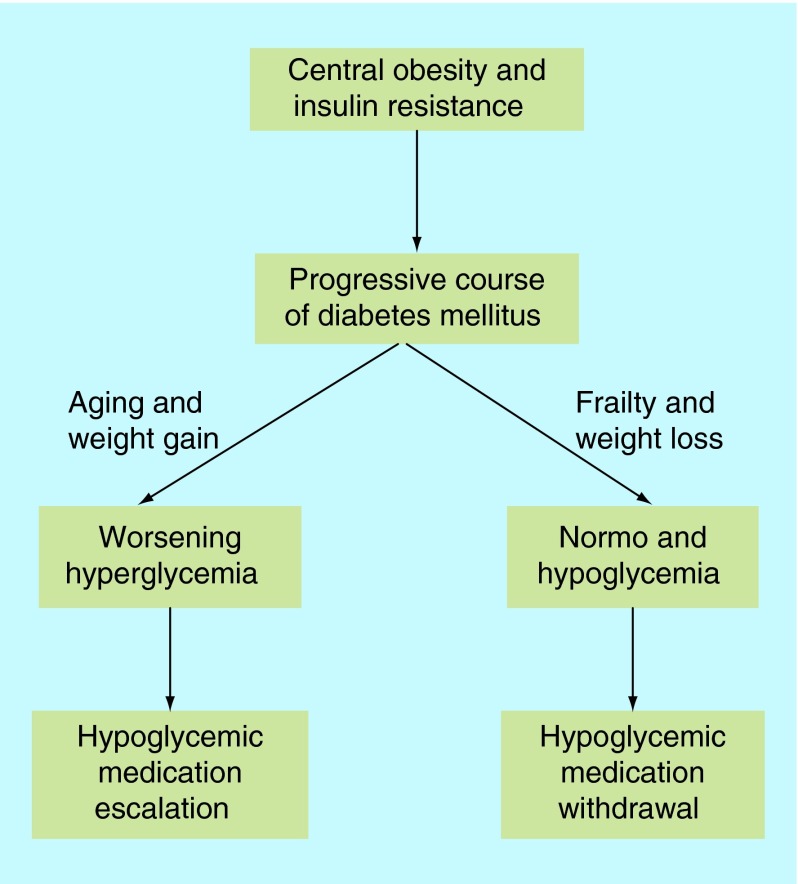
**The emergence of frailty may alter the natural history of diabetes from a progressive to a regressive course leading to downregulation or withdrawal of hypoglycemic medications.**

The prevalence of diabetes is rising with increasing age. In France, the prevalence has increased to 14.2% in those aged 65–74 years, peaking at 19.7% in men and 14.2% in women aged 75–79 years [1]. In the US, total diabetes prevalence is estimated to be 14% of the population and is highest in those aged ≥65 years and by the year 2050 diabetes prevalence could be as high as 33% of the whole population [2]. Diabetes is particularly disabling in older people due to the emergence of geriatric syndromes including frailty in addition to the traditional micro and macrovascular complications. In younger patients, Type 2 diabetes often occurs in the context of obesity and associated insulin resistance. Therefore, treatment in younger age is usually based on lifestyle modifications such as weight loss and progressive drug therapy to achieve glycemic control. However, aging is associated with a marked decrease in bodyweight and food intake [3]. Therefore, in frail older people with Type 2 diabetes, declining body function associated with weight loss and malnutrition may lead to normoglycemia, increased risk of hypoglycemia and reduced body needs of hypoglycemic medications suggesting that a therapeutic approach to reduce or even withdraw hypoglycemic medications in those frail population may be appropriate. This article reviews the impact of frailty on normalization of blood glucose levels increasing the risk of hypoglycemia and the clinical implications of frailty on the management plan of frail older people with Type 2 diabetes.

## Methods

We have performed a search of MEDLINE and Embase from January 1969 to November 2015 using keywords relating to frailty, diabetes mellitus, management, older people, insulin sensitivity, insulin resistance and glucose/insulin dynamics. Only English language articles were selected. Articles were reviewed for relevance by abstract. A manual review of citations in retrieved articles was performed in addition to the electronic literature search. The final list of cited references was chosen on the basis relevance to the topic of review.

## Effect of diabetes on frailty

Older people with diabetes are complicated by various factors such as metabolic dysfunction, vascular disease and the aging process in combination with increased prevalence of geriatric syndromes in terms of cognitive and physical dysfunction. Eventually this complex phenotype leads to malnutrition, physical inactivity and eventually to the development of frailty in some older people with diabetes. Frailty is a condition characterized by increased vulnerability, a reduction in the physiological reserve and compromised ability to resist physical or psychological stressors [4]. Its definition is largely based on the presence of three or more phenotypes (weight loss, weakness, decreased physical activity, exhaustion and slow gait speed) (Figure 1) [5]. Frailty is regarded as a wasting disease with weight loss due to under nutrition predominantly. Under nutrition increases the risk of frailty by almost fourfold (odds ratio [OR]: 3.7; 95% CI: 1.4–9.9) [6]. When frailty happens to older people with diabetes, sarcopenia or muscle mass loss seems to be accelerated. Diabetes increases the risk of muscle mass loss by twofold in older people (≥65 years of age) compared with those without diabetes. Reduction in muscle protein synthesis due to lower testosterone and IGF1 and increase in muscle protein breakdown due to a higher rate of inflammation may be the underlying factors [7]. Sarcopenia due to diabetes may be explained by the increased catabolic rate induced by insulin deficiency and the increased accumulation intramyocellular lipid [8]. Diabetes also increases rate of muscle mass loss and reduces muscle strength leading to a reduction in muscle function and longer sit to stand time compared with those without diabetes [9]. Oral health of older people with diabetes contributes to the malnutrition state and progresses toward frailty. Poor dentition, dry mouth, reduced taste sensation, palatability and appetite change with increasing age are all associated with suboptimal nutritional state [10].

## Effect of frailty on diabetes

Frailty is characterized by weight loss that may increase insulin sensitivity and improve glucose tolerance due to loss of visceral fat. Therefore, with the emergence of frailty insulin/glucose/dynamics may shift in a direction that favors less hyperglycemia (Box 1).

### Normoglycemia

The term ‘burnt-out diabetes’ is used to describe a condition when chronic diseases associated with protein energy malnutrition, muscle wasting and frailty may lead to spontaneous resolution of hyperglycemia and normalization of HbA1c levels [11]. A study in the USA showed that about a third of patients with diabetes and end-stage renal disease (ESRD) on hemodialysis have a normal to low HbA1c level. In this 2-year cohort study of 23,618 patients with diabetes on hemodialysis, 33% of them had HbA1c levels <6% [12]. Lower HbA1c, especially if <5%, was associated with poor survival. It is still not clear what differentiates such patients with normoglycemia or ‘burnt-out diabetes’ from other older people with diabetes who continue to display hyperglycemia but a suggestion that increased frailty in these patients might have a contributing role. About 18–75% of patients with ESRD on maintenance dialysis has malnutrition, muscle wasting or sarcopenia [13,14]. Frailty is strongly associated with all stages of chronic kidney disease (CKD) particularly in patients with moderate to severe CKD and the odds of frailty are inversely related to the estimated glomerular filtration rate (eGFR). Frailty occurs in 20.9% of those with eGFR <45 ml/min/1.73 m^2^. There is approximately twofold increased risk of frailty in mild CKD and approximately sixfold in persons with moderate to severe CKD and it affects up to two-thirds of patients with ESRD on dialysis therapy [15,16]. Frailty increases with aging. This study ascertained that the proportion of frailty gradually increased with age in patients with ESRD on dialysis, from 44.4% in patients younger than 40 years to 66.4% in patients 50–60 years of age to 78.8% in patients over 80 years [16]. Therefore, chronic wasting diseases that lead to frailty may alter the natural history of diabetes from a progressive into a regressive course. Normoglycemia in CKD could be partially explained by renal-specific causes such as reduced renal gluconeogenesis due to renal failure, but CKD and ESRD shared many of the same clinical manifestations as advanced age in the absence of kidney disease, such as inactivity, loss of muscle mass, comorbid conditions and diminished physical and cognitive functions which have been identified as important contributors to frailty [17]. Chronic-wasting conditions are related with increased inflammation and oxidative stress promoting functional decline, accelerated aging and increased adverse outcomes [18]. A study highlighted that the beneficial outcomes of reducing glycemia declines when frailty develops supports the theory of hyperglycemia tends to diminish by frailty. In a cohort study of 2415 older veterans with Type 2 diabetes, mean (standard deviation [SD]) age 73.7 (5.3) years, metformin compared with sulfonylurea, was associated with a 30% decreased risk of mortality among those without any frailty-related diagnoses but was not significantly associated with decreased risk of mortality among those with frailty-related markers after a mean (SD) 5.6 (2.3) years of follow-up. The hazard ratio (HR) for metformin versus sulfonylurea use was 0.69 (95% CI: 0.60–0.79; p < 0.001) in patients without frailty and 0.92 (95% CI: 0.90–1.31; p-value = 0.19) in those who were frail. This suggests that the beneficial effect of metformin on reducing hyperglycemia and subsequently reducing mortality was attenuated in frail older people with diabetes [19]. There is some suggestion that frailty increases insulin resistance and therefore increases hyperglycemia but this has not been confirmed in large clinical trials. The Women’s Health and Ageing Study II demonstrated dysregulation of blood glucose and insulin in response to oral glucose tolerance test in 73 community dwelling women aged 84–95 years not known to have diabetes. Both glucose and insulin responses were more exaggerated and prolonged in the frail versus non frail or pre-frail women [20]. However, this study was limited by small study size and did not provide explanation about the abnormal glucose-insulin dynamics associated with frailty. Furthermore, the frail group in this study, paradoxically, were significantly more obese than the nonfrail group (BMI: 28.4 vs 24.5; p = 0.01, respectively) [20]. Another study showed that insulin resistance increased in frail older people only when abdominal obesity is present while insulin resistance is the same in nonobese frail compared with healthy older persons [21]. Also frailty in some patients with diabetes is associated with weaker muscle strength rather than less muscle mass which is responsible for less insulin sensitivity [22].

### Hypoglycemia

Hypoglycemia may cause frailty to develop and frailty may lead to hypoglycemia setting a vicious circle. The effects of hypoglycemia in older people with diabetes include increased morbidity, frailty, disability and poor quality of life [23]. Impaired autonomic response in older people with diabetes may cause recurrent undetected hypoglycemic events and that leads to cognitive impairment and frailty. Recurrent hospital admissions induced by hypoglycemia has negative effects on older people with diabetes leading to further deterioration in cognitive and physical function. On the other hand, frailty, old age and polypharmacy are associated with increased risk of hypoglycemia. Persons aged ≥80 years had a higher risk (RR: 1.8; 95% CI: 1.4–2.3) of hypoglycemia and those on ≥5 medications (RR:1.3; 95% CI: 1.1–1.5) compared with younger patients or to those on less medications [24]. Older people with diabetes with multiple medications normally have multiple comorbidities and recurrent hospitalization and these are factors that may explain underlying frailty which subsequently linked to higher risk of hypoglycemia [24]. Cognitive dysfunction or dementia is also linked to hypoglycemia risk. It has been shown that the risk of hypoglycemia doubled in older people with combined diabetes and dementia compared with those with diabetes alone [25]. Interestingly hypoglycemic events occurred in older people with diabetes who were not taking any hypoglycemic medications [26,27]. This phenomenon could be related to malnutrition and the effect of frailty in these patients. The excess mortality observed in the intensive glucose control arm in the ACCORD study was not directly explained by the high rate of hypoglycemia [28]. Analysis of the ADVANCE study showed that severe hypoglycemia contributes to adverse outcomes but indicates that hypoglycemia is likely to be a marker of vulnerability rather than a cause of such events [29]. Another study of a cohort of 1342 older patients with diabetes, mean (SD) age 73.3 (5.5) years, demonstrated that multidimensional impairments increased with hypoglycemic events, measured by the multidimensional prognostic index (MPI) score suggesting that hypoglycemia is a predictor of frailty [30]. Data of this study highlighted that the prevalence of hypoglycemic events is significantly higher in patients included in the moderate/severe MPI risk groups who are frailer compared with patients included in the mild MPI risk group. Interestingly, high BMI emerges to be inversely correlated to the multidimensional impairment confirming our suggestion that frailty tend to burn-out diabetes in those who have significant weight loss.

### Mortality

Frailty is likely to be an important factor in studies which showed a relationship between low HbA1c and mortality [31]. As frailty develops, due to under nutrition and weight loss, this will lead to hypoglycemia, lower HbA1c value and less hypoglycemic medications needed. Few studies of patients with Type 2 diabetes which showed a linear relationship between HbA1c and mortality, but without an increased risk in the lowest HbA1c categories. This is likely due to the design of studies that have enrolled younger patients with a shorter duration of diabetes and a lower comorbidity burden [32,33]. By contrast, patients in the studies which showed an increased risk of mortality in the lower HbA1c categories were consistently older age and had more prevalent comorbidities than patients in the higher HbA1c categories (Box 2) [34–43]. Tight glycemic control is not the sole causative factor for poor outcome with hypoglycemia or low HbA1c as hypoglycemia also been observed in patients without diabetes [44,45]. Although studies have adjusted for the associated comorbidities, it is still not clear whether there is an underlying residual biological process that could explain the association between low HbA1c and all-cause mortality. There is hypothesis that patients in the lower HbA1c categories who showed high mortality may have underlying unmeasured factors such as frailty. Frailty was not directly measured in these studies, but markers of inflammation such as elevated ferritin level and markers of malnutrition and weight loss such as low cholesterol and low serum albumin were prevalent in patients with low HbA1c which may suggest poor general health and underlying frailty. Hence, the risk of mortality in older people with diabetes could be correlated to frailty rather than to diabetes itself. In a cohort of 2305 individuals aged ≥70 years of the Canadian Study of Health and Aging, frailty is the strongest mortality predictor (hazard ratio [HR]: 2.72; 95% CI: 2.34–3.16) after 5 years of follow-up [46]. In overweight or obese (median BMI: 34.0 kg/m^2^; range: 24.8–65.1 kg/m^2^) older people with diabetes (median age: 62 years; range: 51–86 years) low HbA1c (≤6.4%) was not associated with increased risk of all-cause mortality that may suggest that weight loss or frailty could be a crucial contributing factor to mortality demonstrated with low HbA1c in other studies [47]. Spontaneous hypoglycemia in hospitalized patients not known to have diabetes was also associated with greater risk of hospital mortality suggesting that hypoglycemia in such patients is likely to be a marker of poor health and frailty rather than a direct cause of death [48]. Therefore, it is rational to consider low HbA1c as a biochemical marker of frailty and a surrogate marker of ‘burnt-out diabetes’ rather than a direct cause of adverse outcomes.

## Frailty & hypoglycemic therapy

Normoglycemia in frail older patients has also been shown in other studies. Hypoglycemic medications have been safely withdrawn in a cohort of frail nursing home older patients with Type 2 diabetes, mean (SD) age 84.4 (6.8) years [49]. The mean (SD) HbA1c at the point of hypoglycemic medications withdrawal was 5.2% (0.4) and 5.8% (1.1) after 6 months of follow-up [49]. We have previously reported complete withdrawal of hypoglycemic medications in eight patients in the community, mean (SD) age 86.5 (3.2) years attending outpatient clinic without deterioration of their glycemic control [50]. Hypoglycemic medications including insulin were completely withdrawn over 3–6 months due to either recurrent hypoglycemia in six patients or tight glycemic control (HbA1c ≤6%) in the other two patients. During 1 year follow-up after complete withdrawal of hypoglycemic medications HbA1c remained stable with no deterioration of glycemic control. The mean (SD) HbA1c at the point of hypoglycemic medications withdrawal was 6.2% (0.8) and 6.5% (0.7) at 1 year of follow-up. The minimum HbA1c before medications withdrawal was 4.6% and the maximum was 7.9% after 1 year of follow-up. Liver and renal functions were similar at the point of medication withdrawal compared with their levels at the point of introducing diabetes treatment suggesting that recurrent hypoglycemia in these patients was not due to progressive organ dysfunctions. Possible indicators for successful withdrawal of hypoglycemic medications included the presence of significant weight loss and development of multiple comorbidities indicating frailty [50]. Another possibility is the development of dementia with increased risk of hypoglycemia. At the point of hypoglycemic medications withdrawal, 50% of patients were already diagnosed with dementia (Table 1). Dementia is known to increase the risk of hypoglycemia and medications review in this group is warranted [25].

## Clinical implications

Glycemic targets should be individualized taking into consideration individuals’ overall health, presence of frailty and expected life span (Figure 2).

### Fit older people with new onset diabetes

Long-term targets based on HbA1c should be around 53 mmol/mol (7%) for the fit elderly in the community with new onset diabetes similar to younger peoples’ targets as this will likely reduce diabetes complications [51].

### Older people with comorbidities & established diabetes

For older people with established cardiovascular disease, multiple comorbidities and long-standing diabetes a safer target around 58–64 mmol/mol (7.5–8%) is more appropriate as the benefit of tighter glycemic control in this group is not established [31]. The benefits of tight glycemic control are compromised by the presence of multiple comorbidities. In an analysis, there was a reciprocal relationship between the burden of comorbidities and the benefits of tight glycemic control. Around 1–2 points were allocated for each comorbidity, according to severity, to create a mortality index score. In people aged 60–64 years with new onset diabetes, the quality adjusted days declined from 106 (95% CI: 97–117) days to 44 days (range: 38–50) with three additional points in mortality index score and to 8 days (range: 5–10) with seven additional index points [52].

### Frail older people

In frail older people with diabetes short-term targets of daily blood glucose level monitoring are more relevant than long-term targets of HbA1c considering their limited life expectancy. A comfortable day to day target of a random blood glucose between >4 but <15 mmol/l seems to be suitable to avoid the development of symptoms as blood glucose outside this range is likely to be symptomatic [53]. Maintaining blood glucose in this ‘comfort zone’ may insure ‘comfort care’ avoiding extreme blood glucose levels to maintain mental function and general well-being [54]. A HbA1c value of around 64–75 mmol/mol (8–9%) is suitable as higher values (HbA1c >75 mmol/mol; >9.0%) is associated with increased mortality [55]. Also, persistent hyperglycemia should be avoided as it is associated with increased risk of falls and mortality [56,57]. Hypoglycemia, on the other hand, is associated with serious consequences and should be at the core of the therapeutic goals of frail older people with diabetes [58]. The increased risk of hypoglycemia in frail older people is not only related to tight glycemic control but also to the overall health condition and associated multiple comorbidities [59]. The emergence of frailty with significant weight loss and malnutrition may lead to a vicious circle of increased risk of hypoglycemia and worsening frailty. Therefore these high-risk patients with significant weight loss and frailty should be recognized by healthcare professionals and hypoglycemic medications review, downregulation or even withdrawal should be considered (Table 2). For example, short-acting rather than long-acting sulfonylureas and long-acting analogs rather than human insulin are safer. Short-acting insulin analogs can be administered only after meal consumption to reduce risk of hypoglycemia if a meal is missed in patients with irregular eating pattern. With recurrent hypoglycemia in frail patients despite of medication review, total withdrawal of hypoglycemic medications appears to be safe and might decrease the risk of the serious consequences of hypoglycemia. Although current guidelines [60] suggest relaxed glycemic control in frail older people with diabetes, we believe that regular medications review should be undertaken as patients get older with consideration of gradual reduction or even complete withdrawal when frailty and significant weight loss emerge.

## Conclusion

The natural history of Type 2 diabetes is progressive with increasing demands of hypoglycemic medications. Increasing age and weight gain promote the progressive nature of its course. However, in the frail older people, particularly in those with chronic wasting diseases and significant weight loss, blood glucose levels tend to normalize with increased risk of hypoglycemia. Recurrent hypoglycemia in frail older people with diabetes is a marker of vulnerability and hypoglycemic medication review or even complete withdrawal is appropriate in this group of patients (Figure 3).

## Future perspective

Frailty is an emerging complication in older people with diabetes and will need interventions beyond glycemic control. Although it may have a positive impact on reducing hypoglycemic medications’ need due to the significant weight loss, it has an overall a negative effect on the outcome with increased mortality. The effect of frailty on glucose/insulin dynamics and its relation with insulin sensitivity/resistance and possible amelioration of hyperglycemia will need further research. There is a need for studies exploring interventions to delay or prevent frailty and disability in older people with diabetes. Improvement in functional level may be more relevant than glycemic control in this population. The multimodal intervention (resistance exercise training, diet and education) proposed by the MID-Frail study in the frail and pre-frail individuals aged ≥70 years with Type 2 diabetes may improve function and preserve self autonomy [61].

**Table 1.** **Comparison of demographics on and at end of hypoglycemic treatment.**

**Parameter**	**On treatment**	**Treatment withdrawn**	**Difference (95% CI)**	**p-value**
Comorbidities, mean (SD)	4.1 (0.9)	6.8 (1.6)	2.7 (1.3–3.9)	0.002
Patients with dementia	None	4 (50%)	–	
Medications, mean (SD)	6.4 (1.9)	10.1 (2.2)	3.7 (1.4–6.1)	0.01
bodyweight (kg), mean (SD)	88 (22.4)	75.4 (21.2)	12.6 (5.9–19.3)	0.003
Mean (SD) ALT	16.8 (4.3)	18.4 (3.3)	1.6 (2.4–5.7)	0.4
Mean (SD) eGFR	46.9 (10.3)	45.1 (10.8)	1.8 (9.5–13.0)	0.7
Mean (SD) HbA1c	6.2% (0.8)	6.5% (0.7)	0.3% (-1.1–0.5)	0.4

The stable liver and kidney functions suggest that successful withdrawal of hypoglycemic medications was not due to deteriorating organ function.

ALT: Alanine transaminase; eGFR: Estimated glomerular filtration rate SD: Standard deviation.

Data taken from [50].

**Table 2.** **Weight loss as a determinant of hypoglycemic medication withdrawal.**

**Patients**	**First weight (kg)^†^**	**Second weight (kg)**	**First HbA1c (%)**	**Second HbA1c (%)**
1	100	70	6.3	6.5
2	95	88	7.1	6.6
3	58	50	6.0	6.8
4	74	64	7.4	7.9
5	127	114	6.0	6.6
6	102	94	5.8	6.1
7	66	60	4.6	5.3
8	82	64	6.3	6.3

First HbA1c = HbA1c at the time of medications withdrawal, second HbA1c = HbA1c after 1 year follow-up off medications. The significant weight loss appears to contribute to the successful withdrawal of hypoglycemic medications.

^†^First weight = peak weight during treatment, second weight = weight at the time of medications withdrawal.

Data taken with permission from [50].

**Box 1. **  **Frailty–diabetes interaction.**

**Effect of diabetes on frailty**

Increased sarcopenia
Decreased muscle protein synthesis
Increased muscle protein breakdown
Increased intramuscular lipid accumulation
Reduced muscle quality
Reduced muscle strength

**Effect of frailty on diabetes**

Less hyperglycemia
Promotes normoglycemia
Increased hypoglycemic tendency
Change natural history of diabetes to a regressive course
Alter insulin/glucose dynamics
Promotes a state of burnt-out diabetes
Increased mortality

**Box 2. **  **Characteristics of patients with low HbA1c and increased mortality.**

Older age
More prevalence of:
  – Anemia
  – Chronic kidney disease
  – Low cholesterol
  – High ferritin
  – Dementia
  – Malignancy
Longer duration of diabetes
More likely to be insulin treated
More likely to have low blood pressure and abnormal liver function tests
Higher level of lung, liver and heart diseases
Lower bodyweight
Higher markers of malnutrition and inflammation
Higher smoking rate
Higher cardiovascular comorbidities

Data taken with permission from [34–43].

**Executive summary**

The natural history of Type 2 diabetes is progressive with increasing hyperglycemia over time requiring escalation of hypoglycemic medications.
With the emergence of frailty and weight loss the natural history of Type 2 diabetes shifts from a progressive into a regressive course leading to normalization of blood glucose levels and increased risk of hypoglycemia.
Hypoglycemic medications review or withdrawal is appropriate in frail older people with recurrent hypoglycemia.
